# Supplementary material for: Polyketide synthase gene clusters in the ericoid mycorrhizal fungus Oidiodendron maius: genome-wide prediction, expression profiling, and functional characterization of knockout mutants
Source: Front Plant Sci. 2026 Sep 16;17:1924430. doi: 10.3389/fpls.2026.1924430 (PMC13623903; doi:10.3389/fpls.2026.1924430)
Supplement: Supplementary file 1 [file SupplementaryFile1.zip › Supplementary Materials/Supplementary Folder 1.zip!/Oidma1_AssemblyScaffolds_Repeatmasked/knownclusterblast/cluster30/jgi.p_Oidma1_44059_mibig_hits.html]

| MIBiG Protein | Description | MIBiG Cluster | MiBiG Product | % ID | % Coverage | BLAST Score | E-value |
| --- | --- | --- | --- | --- | --- | --- | --- |
| EHN79427.1 | alpha-glucosidase\_ScatE1 | BGC0000804 | Acarviostatin\_biosynthetic\_gene\_cluster | 31 | 82.56 | 242 | 1.4e-63 |
| ABC87503.1 | alpha-1,\_4-glucosidase | BGC0001011 | Meridamycin\_biosynthetic\_gene\_cluster | 29 | 80.16 | 204 | 3.2e-52 |
| EHN79423.1 | alpha-amylase\_ScatZ2 | BGC0000804 | Acarviostatin\_biosynthetic\_gene\_cluster | 29 | 36.64 | 82 | 2.4e-15 |
